# Recent Advances in Sensing Applications of Molecularly Imprinted Photonic Crystals

**DOI:** 10.3389/fchem.2021.665119

**Published:** 2021-06-14

**Authors:** Jing Fan, Lili Qiu, Yu Qiao, Min Xue, Xiao Dong, Zihui Meng

**Affiliations:** ^1^School of Chemistry and Chemical Engineering, Beijing Institute of Technology, Beijing, China; ^2^School of Design and Arts, Beijing Institute of Technology, Beijing, China

**Keywords:** photonic crystals, molecular imprinting, sensors, environmental pollutants, human health

## Abstract

Photonic crystals (PhCs) with a brightly colored structure are novel materials and are widely used in chemical and biological sensing. Combining PhCs with molecular imprinting technology (MIT), the molecularly imprinted PhC (MIPC) sensors are fabricated, which can specifically recognize the target molecules. Aside from high sensitivity and selectivity, the MIPC sensors could recognize the naked eye detection because of its optical properties. In this review, an overview of recent advances in sensing applications of MIPC sensors including the responsive mechanisms, application in environmental monitoring, and the application to human health were illustrated. The MIPC sensors all responded to the analytes specifically and also showed high sensitivity in real samples, which provided a method to realize the rapid, convenient, naked eye, and real-time detection. Furthermore, the current limitations and potential future directions of MIPC sensors were also discussed.

## Introduction

With the development of analytical methods, many technologies have realized real-time detection with high selectivity and sensitivity. Gas chromatography–mass spectroscopy (GC–MS) is commonly used to detect the concentration of volatile targets, with low limit of detection (LOD) and limit of quantitation (LOQ) (Jung et al., 2021; Naik et al., 2021; Tian et al., 2021; Vollmer et al., 2021). Ion mobility spectroscopy (IMS) is excellent at detecting explosive gases and is now widely used in airport security checks (Gaik, 2017; Hagan, 2017; Dai et al., 2018; Horestani, 2018; Shahraki et al., 2018). The electrochemical sensor converts chemical signals into electrical signals, which have the advantages of quick detection, high sensitivity, and easy modification (Benny et al., 2021; Carneiro et al., 2021; Chu et al., 2021; Li et al., 2021). However, due to the professional operation, strict measurement conditions, expensive equipment, and others, the application of these technologies is greatly limited. An ideal sensor is expected to respond quickly and intuitively to the kind and concentration of the object without any other auxiliary equipment. Based on these, photonic crystals (PhCs) have become the ideal sensing material for naked eye detection by adjusting its structural color in a specific environment through the design of its chemical components. Compared with traditional biochemical sensors, PhCs sensors have shown broad application in the fields of rapid screening and point-of-care diagnostics with the label-free, visual, and on-site detection (Chen J. Y. et al., 2019; Kou et al., 2019; Snapp et al., 2019). Yan et al. (2019) reported inverse opal silk methylcellulose PhC films (SMPCF), which were fabricated through a traditional self-assembly method, and displayed an excellent sensing performance with instantaneous color changes from green to red when alternately exposed to organic solvents.

Moreover, PhCs are combined with many technologies to improve its selectivity and sensitivity, such as surface plasmon resonance (SPR) (Gupta et al., 2016; Huang et al., 2020; Islam et al., 2020; Wang et al., 2020), surface-enhanced raman scattering (SERS) (Beffara et al., 2020; Dedelaite et al., 2020; Kraai et al., 2020; Wang et al., 2021), etc. Molecular imprinting technology (MIT) when combined with PhC sensor can be used to create biomimetic materials with specific recognition sites (Huang S. Y. et al., 2018; Zhao et al., 2018). Qiu's (Qiu et al., 2020) group prepared a molecularly imprinted PhC (MIPC) and detected alpha-amanitin. The prepared MIPC sensor possessed a wide linear range (10^−9^–10^−3^ mg/L), changed in visual color, had a low detection limit (10^−10^ mg/L), had a short response time (2 min), and had good reusability. As an antibody simulation technology, MIT has been widely used to improve the selectivity and sensitivity of sensing materials because of its high mechanical strength and flexibility (Capoferri et al., 2018; Hua et al., 2018; Mo et al., 2019).

Here, we aim to provide an overview of the application of MIPC sensors. We review the MIPC sensors in three aspects, including responsive mechanisms, application in environmental monitoring, and application in human health. At the same time, the limitations and future directions of MIPC sensors are also discussed.

## MIPC Sensors

### PhCs

The concept of PhCs originated from the research on self-radiation and photon localization by Yablonovitch (1987) in 1987. PhCs are ordered structural materials formed by the periodic arrangement of two or more materials with different refractive indices in space, and the repeated unit is the order of optical wavelength (Breuer-Weil et al., 2016). Due to the periodic structure, PhCs meet the Bragg's diffraction law and have a photonic band gap (PBG) (Sun et al., 2015; Vogel et al., 2015). PhCs have the function of wavelength selection because of the existence of PBG. When a beam of light irradiates on the PhCs, the corresponding bright color of the Bragg diffracted light which is within the visible range will appear on the surface of the PhCs, and this bright color is called “structural color.” By reasonably designing the material composition, effective refractive index and lattice parameters of PhCs, PhCs with specific PBG can be artificially prepared (Brooks and Sumerlin, 2016; Li et al., 2016).

According to the periodic arrangement, PhCs can be divided into one-dimensional (1D), two-dimensional (2D), and three-dimensional (3D) PhCs. 1D-PhCs is the simplest PhCs material, and its refractive index has periodicity in only one dimension. In 1887, Rayleigh (2009) first studied the propagation of electromagnetic waves in periodic media, which was related to the special reflection properties of crystalline minerals with “twining” periodic. It was found that 1D-PhCs had a narrow band gap, which prohibited light transmission and broadcasting on the plane. This band gap was related to the angle, which was due to the fact that the light went through different cycles in the case of an abnormal incident, and the resulting reflected color changed sharply with the angle (Zhao et al., 2012). Over the next century, multilayers were studied, but it was not until 100 years later that Yablonovitch and John introduced the concept of 2D and 3D omnidirectional PBG.

The first artificial PhCs structure was fabricated using a mechanical drilling method by Yablonovitch and Gimmter in 1989. Then, in 1991, Yablonovitch improved the method and produced the first 3D-PhCs with a full PBG. At the beginning of the study, the mechanical drilling method was successfully used to prepare PhCs. However, the process was complicated and the preparation period was long, and it was seldom used in the current research and preparation. With the development of technology, more preparation methods have been rapidly developed. Layer-by-layer lithography is one of the most common methods. The PhCs prepared through this method have a complete structure, have fewer defects, and are widely used in optical fibers and optical calculation machines, chips, and other optical fields (Yablonovitch et al., 1991). This method has high requirements for equipment and lacks dynamics adjustability. It is also difficult to popularize and apply in the field of material science. At present, the simpler method for PhCs fabrication is the colloidal self-assembly due to its low cost and easy functionalization. In this method, long-range ordered nanostructures are formed of monodisperse colloidal particles through a colloidal self-assembly process.

PhCs with bright structure color are also found in nature, such as the feathers of birds and the wings of butterflies, and many plants (He et al., 2018). Many animals with PhCs structure will change their color according to their to camouflage themselves or as a warning to their enemies (Mäthger et al., 2003; Liu et al., 2009). Inspired by the discoloration of natural PhCs, this ordered materials are used in colorimetric sensors, which can directly convert external stimuli into visible color changes with a low-cost and simple operation (Lai et al., 2012; Frascella et al., 2013). At present, PhCs sensors have been applied to the detection of humidity (Kou et al., 2018; Di Palma et al., 2019), organic solvents (Yu et al., 2020), antibiotics (Hou et al., 2015), and so on (Wu et al., 2019; Qiu et al., 2020; Rizvi et al., 2020).

### MIT

MIT is the technology of preparing polymer with selectivity for a specific target molecule, which is called the template molecule or imprinted molecule. Dickey first proposed the concept of “molecular imprinting” in 1949 (Dickey, 1949); however, due to the limited experimental materials for this technology, it did not attract the attention of the world for a long time. It was not until Wulff (1995) first synthesized molecularly imprinted polymers (MIPs) with specific recognition properties for glyceric acid and its enantiomers in 1972 that a breakthrough was made in MIT. Later in 1984, Mosbach introduced the non-covalent binding between a template and functional monomer into the synthesis of molecular imprinting polymers (MIPs) (Andersson et al., 1984), and carried out a series of extended work (Kempe and Mosbach, 1995). Especially in the 1990s, Mosbach published theophylline-MIPs synthesized by non-covalent method in *Nature* (Vlatakis et al., 1993). At the same time, the Society for Molecular Imprinting (SMI) was founded in Sweden, which comprehensively promoted the rapid development of MIT and made it the hotspot of research. In recent years, MIT is mainly involved in the fields of polymer synthesis, materials chemistry, and so on. The research on the imprinting mechanism, the preparation of MIPs, and the thermodynamics and kinetics have been rapidly developed.

In the preparation of MIPs, template molecules interact with functional monomers to form supramolecular compounds, and the polymer is formed under the action of cross-linking agent. After removing the template molecules by physical or chemical methods, MIPs contain binding sites complementary to the spatial shape and the functional group positions of template molecules are obtained (Chen et al., 2012). According to the different binding modes between template molecules and functional monomers, MIT can be divided into covalent imprinting, non-covalent imprinting, semi-covalent imprinting, and metal complexing imprinting.

Covalent imprinting was first proposed by Wulff in the 1970s. Under the action of a suitable crosslinker and initiator, the template molecule binds to the functional monomer by a covalent bond, which is reversible. Then, using chemical methods to break the covalent bond of the complex, the MIP with a specific recognition site is obtained. The MIPs formed by covalent imprinting have high selectivity and recognition ability. However, due to the strong binding ability, it is difficult to remove the template molecule, which limits the application and development of this method. Non-covalent imprinting refers to the formation of stable prepolymers between monomers and template molecules under the interaction of non-covalent bonds such as Van der Waals forces, hydrogen bonds, and hydrophobicity, and the polymerization is obtained under the action of cross-linking agents and initiators (Arshady and Mosbach, 1981). The non-covalent imprinting is simple in preparation and the template becomes easy to remove. However, the selectivity of MIPs made by non-covalent imprinting is low. Semi-covalent imprinting combines the advantages of covalent and non-covalent methods. Templates form polymers with functional monomers through covalent interaction, and non-covalent bonding is used for detection during the adsorption process. The method of metal complexation is mainly based on chelation between metal ions and molecules.

With the development of MIT, a variety of preparation methods of MIPs have been developed, which can be divided into bulk polymerization, precipitation polymerization, *in situ* polymerization, suspension polymerization, and surface imprinting. Bulk polymerization is the earliest and is one of the most commonly used methods at present. Each component of the polymer is dissolved in a solvent, and the MIPs were obtained after heating or photoinitiation with oxygen-free conditions, which need to be ground and screened before use. Precipitation polymerization is a heterogeneous solution polymerization method. Each component is dissolved in the pore-forming agent to form a polymer that is highly cross-linked, and the resulting polymer is not soluble in the pore-forming agent to form a precipitate. *In situ* polymerization is usually used in the preparation of a monolithic column or imprinted film. The reaction components are packed into a chromatographic column or capillary column with a certain proportion to get imprinted polymer, which can be directly applied to the enrichment and extraction of chromatography conveniently and practically. Suspension polymerization is commonly used to fabricate molecularly imprinted microspheres. Generally, hydrophobic solvent is used as the dispersed phase, and water or organic solvent with high polarity is used as the continuous phase. After adding continuous phase with violent stirring and nitrogen condition, the dispersed phase disperses to form small droplets for a polymerization reaction. Surface imprinting is a new method, which is usually used to modify the surface of a silica gel to carry active groups such as an amino group. This method reduces the occurrence of embedding phenomenon and has a good elution effect. As the modification groups are bound by covalent bonds, the recognition of target molecules is more specific.

MIT has been widely used in many fields such as food, environment, and medicine due to its simplicity, strong stability, and high selectivity. It has also shown promise in separation applications, catalysis, membrane separation, and sensors (Kou et al., 2012; Meng et al., 2015; Chen et al., 2016; Fizir et al., 2020).

### Responsive Mechanisms of MIPC Sensors

The diffraction wavelength and structure color changes of PhCs follow Bragg's law, and the maximum diffraction light wavelength λ_max_ meets Equation (1):

(1) $$\begin{array}{r} \lambda_{\text{max}}=1.633\left(\text{d}/\text{m}\right){\left(n_{a}^{2}-{\text{sin}}^{2}\theta \right)}^{1/2} \end{array}$$

Where d is the diameter of particles and m is the Bragg diffraction order. The n_a_ is the average refractive index of the composed material of PhCs and θ is the angle between the incident light and the normal vector of PhCs plane. The *n*_a_ can be calculated by:

(2) $$\begin{array}{r} {\text{n}}_{\text{a}}={\left[{\text{n}}_{1}^{2}\text{f}+{\text{n}}_{2}^{2}\left(1-f\right)\right]}^{1/2} \end{array}$$

*n*_1_ is the refractive index of composed particles and *n*_2_ is air. Linking Equations (1) and (2), λ_max_ depends on *d* and *n*_a_ when θ is fixed.

**Figure 1:**
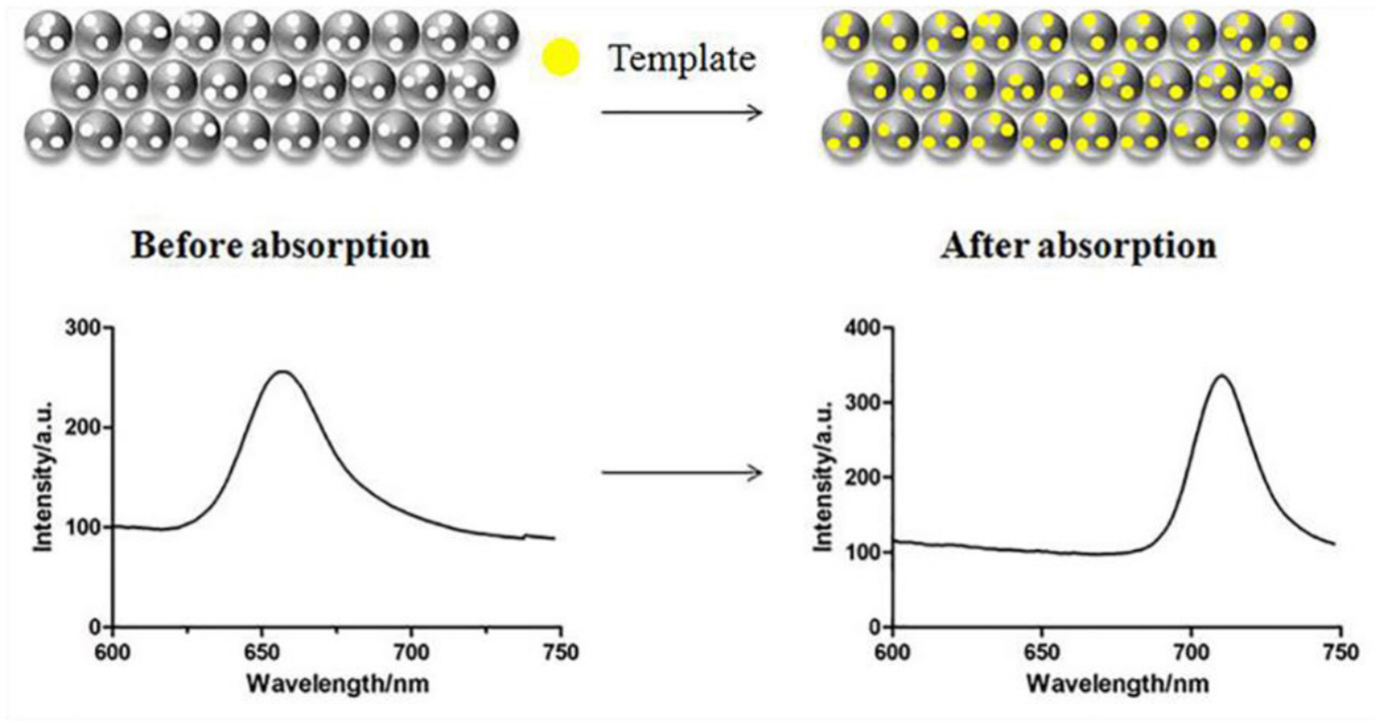
The response mechanism of molecularly imprinted photonomic crystals.

In the process of molecular recognition, when the refractive index of the target molecule is different from that of the MIPC, *n*_a_ will change. At the same time, after binding with the target molecule, the MIPC will either shrink or swell due to the change in osmotic pressure, resulting in the change in *d*. In short, MIPC sensors shrink or swell when the target molecules are bound with the specific sites on MIPC sensors, which will cause changes in *d* and *n*_a_ of MIPC sensors. Subsequently, the changes cause the shift of λ_*max*_ as shown in Figure 1 shows. If λ_*max*_ appears in the visible or near-infrared light region, the change of structure color caused by λ_*max*_ shift can be observed by naked eye, which achieves the purpose of “naked eye detection” (Han et al., 2012; Lai et al., 2015).

The combination of MIPs and PhC sensors is a great breakthrough in detection technology. In the process of molecular imprinting, the PhC sensors are introduced to the binding site of the target molecule, which the PhC sensors have higher sensitivity and selectivity. Meanwhile, the special optical properties of PhC sensors enable the molecular recognition process to be directly expressed by the optical signals without any additional label (Endo et al., 2013; Zhang et al., 2013). Hu et al. (2006) first proposed a MIPC sensor and applied it to the recognition of chiral compounds. The sensor showed high sensitivity and selectivity to L-3, 4-dihydroxyphenylalanine. Then, they also prepared MIPC sensors for the detection of protein (Hu et al., 2010), atrazine (Wu et al., 2008), theophylline, and ephedrine (Hu et al., 2008), which obtained a good response. Chen et al. (2014) prepared a hollow MIPC sensor to detect hemoglobin. The diffraction wavelength of the sensor red shifted evidently as the structure color changed. Moreover, the MIPC sensor detection for chlorotoxin (Wang et al., 2012), bisphenol A (Guo et al., 2012; Oh et al., 2020; Yang and Park, 2020), diethylstilbestrol (Sai et al., 2013), hormone, (Kalecki et al., 2020) and other molecules (Hong et al., 2020) have also been reported as shown in Table 1.

**Table 1:** Molecularly imprinted photonomic crystals sensors and their target analytes.

**Category**	**Analytes**
Antibiotics	Oxytetracycline (Wang et al., 2019a), clindamycin hydrochloride (Wang et al., 2019b), chloramphenicol (Sai et al., 2016, 2019), sulfonamides (Zhang F. et al., 2018; Zhang Y. H. et al., 2018; Li et al., 2019; Lin et al., 2020)
Organophosphates	Parathion (Zhang X. et al., 2017), methane phosphonic acid (Huang C. et al., 2018), monocrotophos (Hong et al., 2020)
Explosives	TNT (Lu et al., 2016, 2017; Fan et al., 2020), 2,6-DNT, 2,4-DNT (Dai et al., 2017b; Lu et al., 2017) and 4-MNT (Lu et al., 2017), RDX (Fan et al., 2020), HMX (Fan et al., 2020), CL-20 (Fan et al., 2020)
Drug	Dropropizine (Chen Z. et al., 2019), drug delivery (Deng et al., 2018), sulpiride (Zhang et al., 2017)
Proteins and amino acids	Fibrinopeptide B (Resende et al., 2020), avidin (Jinn et al., 2019), S-layer proteins (Pan et al., 2019), glycoprotein (Chen W. et al., 2019; Wang H. et al., 2020), L-kynurenine (Rizvi et al., 2020), L-histidine (Chen et al., 2018), hormone (Dabrowski et al., 2019; Kalecki et al., 2020)
Others	Bisphenol A (Shin and Shin, 2019; Hong et al., 2020; Oh et al., 2020; Yang and Park, 2020; Zeng et al., 2020), 2,4-dichlorophenol (Qin et al., 2020), 2-butoxyethanol (Dai et al., 2017a), α-amanitin (Qiu et al., 2020), ethyl anthranilate (Zhang et al., 2019), benzocaine (Chen S. et al., 2019), testosterone (Kadhem et al., 2018), phthalate esters (Gong et al., 2017), aspartame (Yang and Park, 2018) aloe-emodin (Wang and Kan, 2020), folic acid (Yang et al., 2020)
Metal ions	Ca^2+^ (Dai et al., 2017a), Ni^2+^ (Ravikumar et al., 2019)

## Application in Environment Monitoring

### Antibiotics

Antibiotics have anti-pathogen activities and can interfere with the development of other cells (Gothwal and Shashidhar, 2015). Currently, antibiotics can not only be used as a drug to treat and prevent infectious diseases, but also be widely used in feed additives (Mathew et al., 2007). Contamination with antibiotics has been found in soils, sediments, sludges, underground water, wastewater, tap water, surface water (lakes, streams, rivers, and oceans), plants, and aquatic animals due to the rampant use of antibiotics in medicines and feed additives (Simazaki et al., 2019).

Methods including UV spectrophotometry, thin-layer chromatography (TLC), liquid chromatography–mass spectrometry (LC–MS) (Robert et al., 2015), high-performance liquid chromatography (HPLC) (Cámara et al., 2013), ELISA, and biosensors are used to detect antibiotics. However, complex operation, poor stability, and the high cost limit their better performance in the detection. It is necessary to develop a method for the real-time detection for antibiotics with high sensitivity and selectivity, easy operation, and low cost.

**Figure 2:**
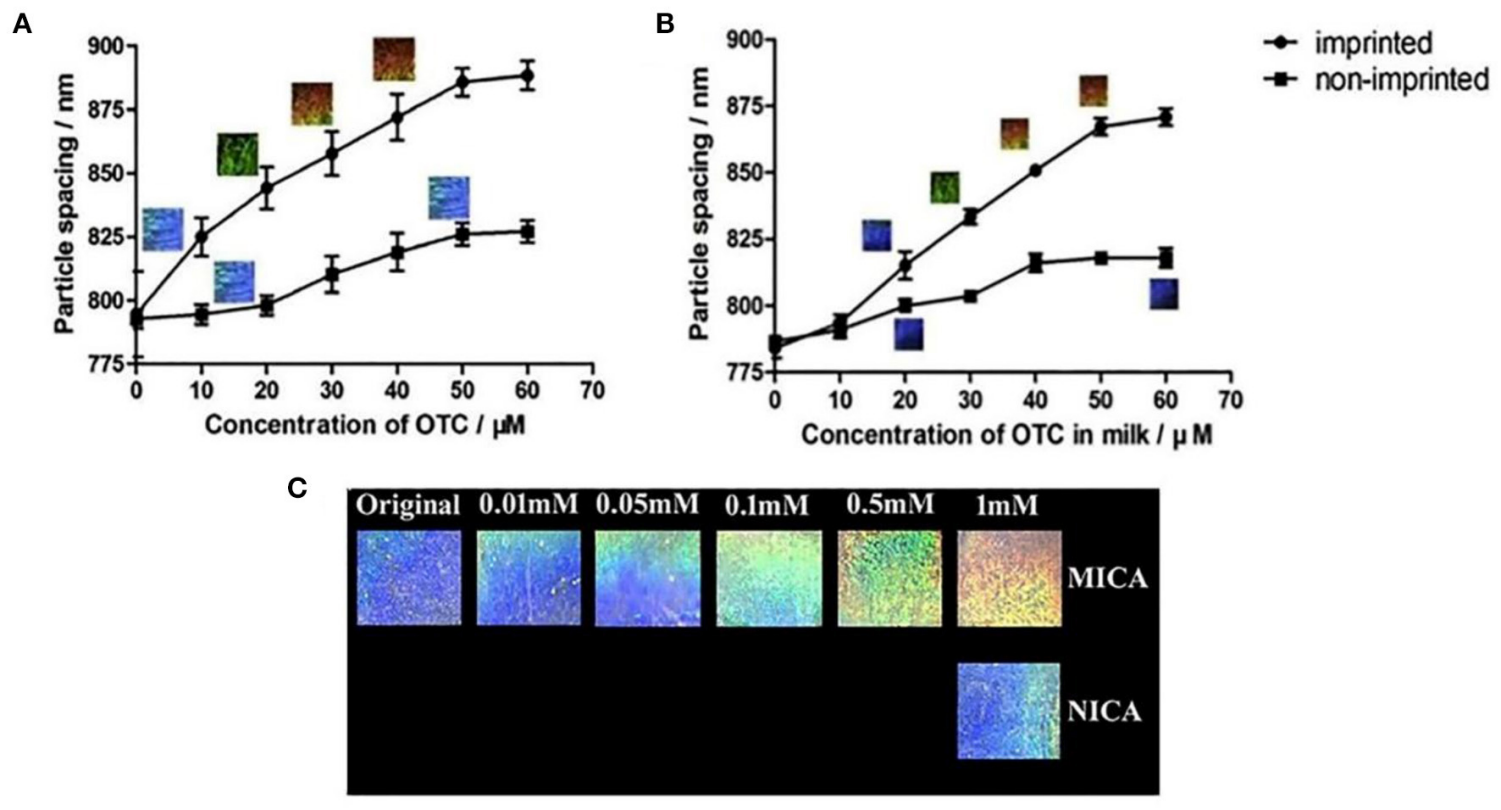
The analysis of oxytetracycline (OTC) by OTC-imprinted hydrogels and non-imprinted hydrogels in **(A)** aqueous solution and **(B)** milk, respectively (reprinted with the permission of Wang et al., 2019a; copyright 2019 Elsevier); **(C)** the structure color change of molecularly imprinted colloidal array and non-imprinted colloidal array (NICA) in clindamycin hydrochloride solution (reprinted with the permission of Wang et al., 2019b; copyright 2019 Royal Society of Chemistry).

Wang et al. (2019a) created a molecularly imprinted two-dimensional PhC hydrogel (MIPCH) sensor for the fast screening of tetracyclines (TCs) in milk. TCs belong to the broad-spectrum antibiotics and are often used to cure the acute diseases caused by the Gram-positive and Gram-negative bacteria, which are extensively used as feed additives to control bacterial infections and to promote animal growth in animal husbandry. The hydrogel was imprinted with oxytetracycline (OTC) and the response was monitored through a readable change of Debye diffraction ring, which is related to the particle spacing of MIPCH. As the concentration of OTC increased from 0 to 60 mM, the particle spacing of MIPCH sensor increased to about 94 nm and the structural color redshifted from blue to red as shown Figure 2A. Also, the non-imprinted hydrogel had a poor response and no obvious structural color change. The OTC-MIPCH showed high selectivity to OTC and the sensing reversibility of MIPCH was over five rounds. The detection of OTC in a milk sample by this portable, cost-effective MIPCH sensor had also been achieved at the same range of OTC. Figure 2B showed that with the increasing concentration of OTC in the milk sample, the particle spacing of MIPCH sensor increased about 92 nm and the structural color of MIPCH changed from blue through green to orange.

Later, Wang et al. (2019b) reported another MIPCH for the detection of clindamycin hydrochloride (CLI) as the same method. The MIPCH showed a significant response to CLI, and the particle spacing shifted 41 nm within 10 min in response to 1 mM CLI, as the structural color changed from blue, green, to yellow, and finally, to red in Figure 2C. These low-cost and naked-eye sensing materials provide a label-free way to detect antibiotics.

Sulfonamides (SAs) are a class of antibiotics with amino phenyl sulfonamide chemical structure and are widely used in animal husbandry and aquaculture due to the broad antibacterial spectrum, cheap price, and high stability (Arsenault et al., 2007; Arroyomanzanares et al., 2014). Li et al. (2019) reported a PhC MIP (PCMIP) for the rapid detection of sulfaguanidine (SG) in fish. The reflection wavelength of PCMIP red shift as the concentration of SG increased, and the relationship between wavelength shift and the concentration was: Δλ = 7.8887 lg (C) + 79.9664. The response was rapid with only 5 min and the LOD of PCMIP was 2.8 × 10^−10^ mol/L. In lake water and fish samples, the recovery rates ranged from 93.8 to 111.2% and from 88.5 to 115.2%, which suggested that the sensor can be used in food samples with complicated matrices.

Subsequently, they reported a four-channel PCMIP sensor array (Lin et al., 2020) as the same methods for the simultaneous detection of various SAs. The array was composed with four units. SG, sulfamethazine (SM_2_), and sulfathiazole (ST) were chosen as template molecules for three units of these as SG-PCMIP, SM_2_-PCMIP, ST-PCMIP, and the other unit was prepared without a molecule as PCNIP. The array was used to analyze six SAs, SG, SM_2_, ST, sulfadiazine (SD), sulfadimethoxine (SDM), and sulfanilamide (SA) at concentrations of 10^−4^,10^−6^, and 10^−8^ mol/L. At the concentration of 10^−4^ mol/L, three SAs-PCMIP responded to their template molecules obviously due to the abundant imprinted sites for corresponding template molecules, and the PCNIP showed the lowest sensitivity and selectivity across six SAs. And the sensor array exhibited the similar response at other concentrations. Principal component analysis (PCA) was used to reduce the above multi-dimensional data to 2D or 3D dataset. PCA reduced the complexity and size of the training data and transformed them into roots that were linear combinations of the response patterns. Two principal components (PC1 and PC2) were extracted after PCA analysis, and the cumulative percentage reached 88%, which indicated that PC1 and PC2 almost contained the total information of the four variables. The PCA plot showed that the signals of six SAs were distinctly distributed, which proved the selectivity of PCMIP method. Linear discrimination analysis (LDA) was also used, and PCMIP six SAs responses in LDA plot of PCMIP at the concentration of 10^−4^ mol/L were clustered into six tight groups, which also indicated that the sensor assay exhibited excellent discrimination for the six SAs. The array was used to investigate the SAs in fish samples, and the LDA plot was shown. The solid ellipses of 1–6 represented the discrimination of the spiked SAs, and the dotted ellipses represented the predicted areas at which the spiked SAs should be. Clearly, the expected areas and the measured areas matched well with a discrimination accuracy of 90.9% in spite of the interference from complex components in the fish matrix. Thus, the as-prepared array proposed a strategy applicable in food analysis.

Zhang F. et al. (2018) fabricated an optical molecular imprinted sulfonamides sensor by nanocrystalline cellulose. A chiral nematic imprinted composite film appeared red was synthesized, and the film shows a naked-eye color response to sulfanilamide, which is related to reassemble imprinted sites in the chiral nematic structure, resulting in a yellow reflecting film. The sensor also responded to three sulfonamides upon exposure to various antibiotics. Because of the special selective binding to the similar spatial configuration of imprinted template, the sensor showed uniquely optical response to three SAs and less light shift of other antibiotics.

### Organophosphates

Organophosphates play a more and more important role in agricultural pharmaceutical production and more than 100 kinds of organophosphates are in use. Under normal circumstances, Organophosphates can be decomposed through various physical, chemical, and biological processes such as hydrolysis, photolysis, and microbial degradation, and can also be rapidly degraded or transformed in the body through a variety of detoxification pathways (Wallace, 1992). However, due to its extensive use in agriculture, organophosphates have become the environmental pollutants.

**Figure 3:**
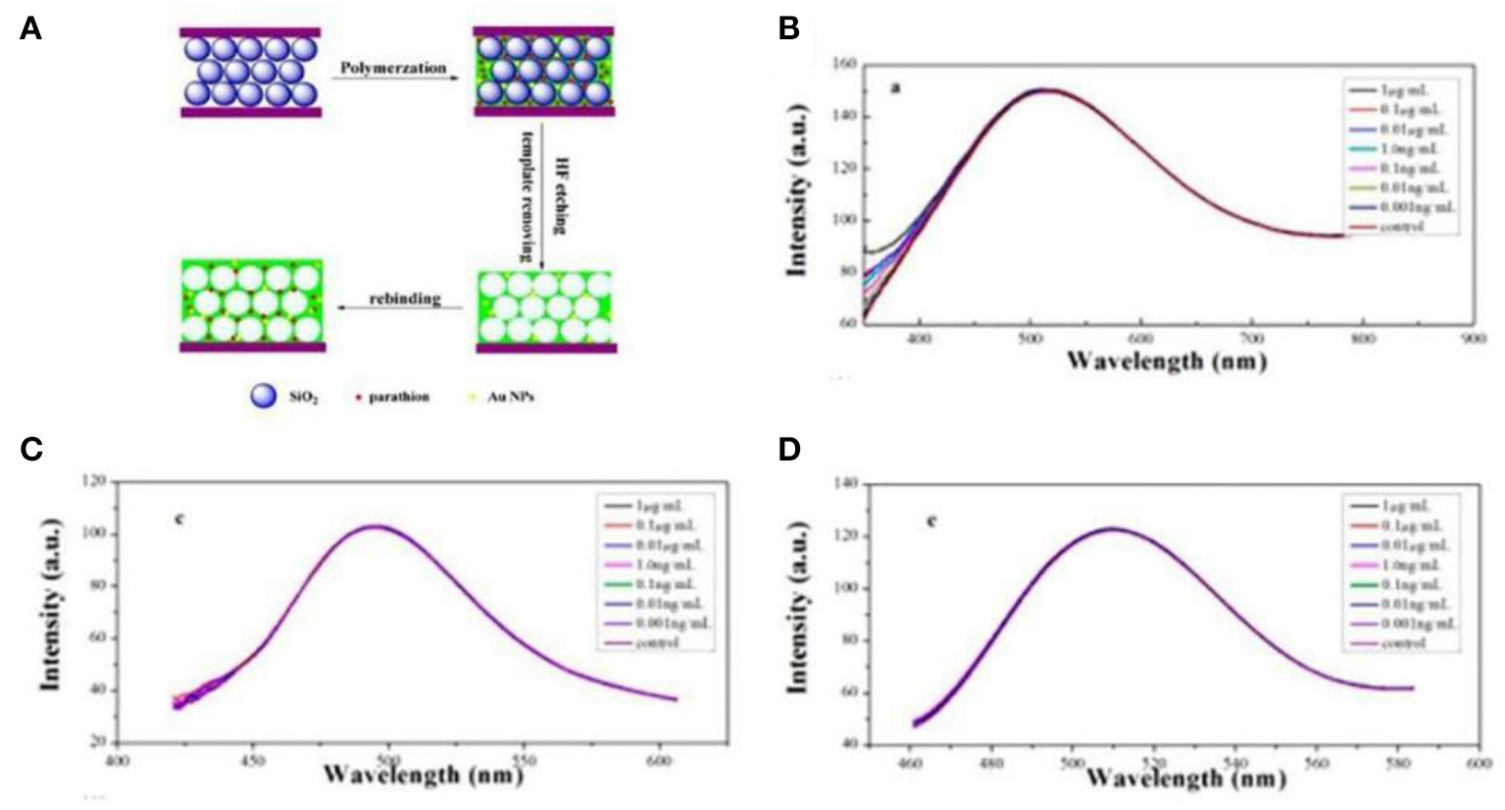
**(A)** The synthetic procedure of fabricated a gold doped imprinted inverse opal photonic crystals (Au MIPs IO PCs); optical responses of Au-MIP IO PCs toward **(B)** methyl parathion, **(C)** monocrotophos, and **(D)** malathion (reprinted with the permission of Zhang X. et al., 2017; copyright 2017 American Chemistry Society).

Parathion is a kind of broad-spectrum pesticide, which could be harmful to the nervous, reproductive, endocrine, and immune systems (Liu et al., 2015; Trinder et al., 2016). Zhang X. et al. (2017) fabricated a gold doped imprinted inverse opal PhC (IO PC) for the fast determination of parathion as shown in Figure 3A. In Figure 3, the selectivity of Au-MIP IO PCs was studied, and three kinds of pesticides, methyl parathion, monocrotophos, and malathion, were selected as analytes. The Au-MIP IO PCs displayed a specific response toward parathion and the selectivity to other competitive pesticide molecules. The response time was only 5 min, and the parathion could be well-detected from real water samples. The recovery rates were between 95.5 and 101.5%.

Huang C. et al. (2018) designed a long-range ordered methane phosphonic acid (MPA) imprinted microporous inverse opal hydrogel particle. The crystal colloidal arrays (CCAs) hydrogel particles can be used as the colorimetric sensors for the detection of the existence of the MPA. The color of CCA particles changed from green to red in the MPA region and the largest red shift was 120 nm. Moreover, the reflection peak shift (Δλ) showed a good linear relationship with the logarithmic distribution of the MPA concentration (c) in the range from 1 × 10^−6^ to 1 × 10^−3^ M. This CCAs hydrogel particles provide the potential application for the building of the pesticide detection system.

### Explosives

Residues in the synthesis or transportation of many explosives or explosive intermediates will cause environmental pollution and endanger human health (Gulati et al., 2019; Peter et al., 2019). Real-time, sensitive, and simple explosive sensor technology is of vital importance in counter-terrorism, public security, and military applications.

Lu et al. (2016) explored a molecularly imprinted colloidal array (MICA) for the selective visual detection of 2,4,6-trinitrotoluene (TNT).

**Figure 4:**
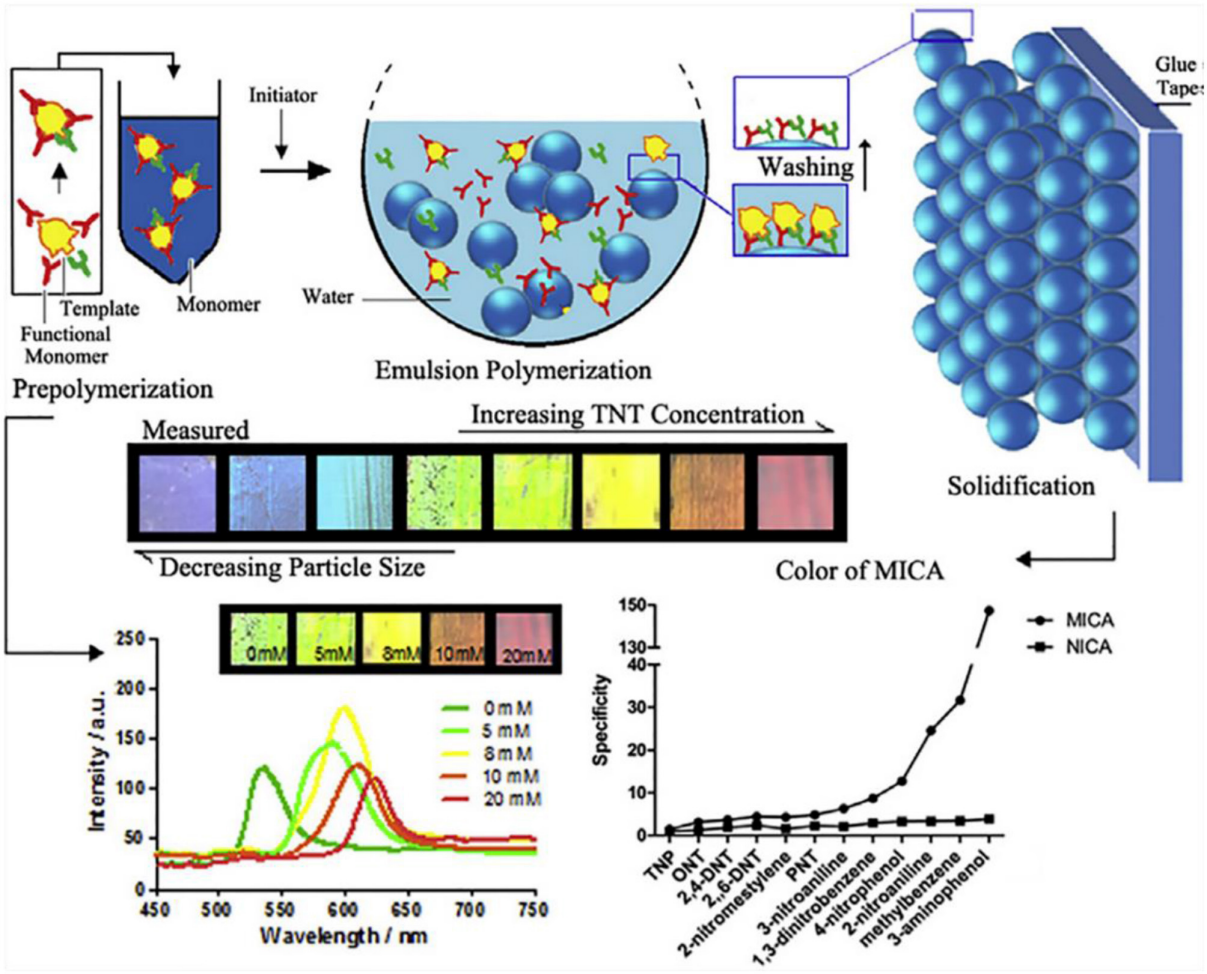
The fabrication of the molecularly imprinted colloidal array and the visual detection of 2,4,6-trinitrotoluene (reprinted with the permission of Lu et al., 2016; Copyright 2016 Elsevier).

Figure 4 showed that the LOD of the MICA sensor was 1.03 μg and the response time was 3 min. The color of the MICA changed from green to red with an 84 nm diffraction red shift as the TNT concentration increased to 20 mM. The sensor was selective for TNT compared with similar compounds such as 2,4,6-trinitrophenol, 2,4-dinitrotoluene,2,6-dinitrotoluene, 2-nitrotoluene, 4-nitrotoluene, 2-nitromesitylene, 1,3-dinitrobenzene, methylbenzene, 4-nitrophenol, 2-nitroaniline, 3-nitroaniline, and 3-aminophenol.

**Figure 5:**
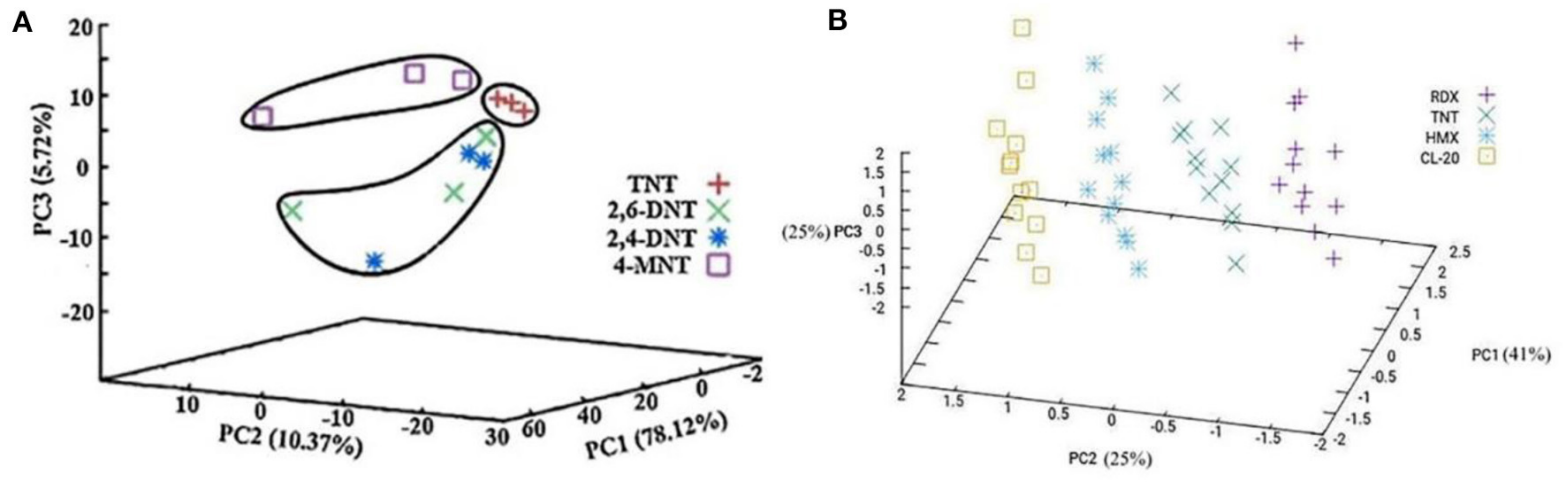
Principal component analysis score plot for **(A)** TNT, 2,6-DNT, 2,4-DNT, and 4-MNT (reprinted with the permission of Lu et al., 2017; Copyright 2017 Elsevier); **(B)** RDX, TNT, HMX, and CL-20 of the first three principal components (reprinted with the permission of Fan et al., 2020; Copyright 2020 Elsevier).

Later, Lu et al. (2017) reported a colorimetric sensor array based on four kinds MICA for the selective visual detection of TNT, 2,6-dinitrotoluene (2,6-DNT), 2,4-dinitrotoluene (2,4-DNT), and 4-nitrotoluene (4-MNT). The single MICA displayed obvious color change from green to red to 20 mM in four explosives with red shifts of 84 nm (TNT), 46 nm (2,6-DNT), 54 nm (2,4-DNT), and 35 nm (4-MNT). Using PCA and pattern recognition (PR), as shown in Figure 5A, the cross-reactive array showed better classification and identification ability, and this novel detection platform provided a wider applicable range among other explosives in a simple sensor array with customized functionality.

Based on this technology, Fan et al. (2020) also fabricated a sensor array to detect four ammonium nitrate explosives, 1,3,5-trinitro-1,3,5-triazacyclohexane (RDX), TNT, cyclotetramethylenete–tranitramine (HMX) and 2,4,6,8,10,12-hexanitro-2,4,6,8,10,12-hexaazaisowurtzitane (CL-20). Every single sensor red shifted in the corresponding explosive with color change, and the sensor array can discriminate four explosives with a LOD of 1 mM. The PCA score plot in Figure 5B showed the response of RDX, HMX, CL-20, and TNT scattered in separate areas. The sensor array provides a naked-eye qualitative and semi-quantitative detection method for explosive detection.

### Other Environmental Pollutants

**Figure 6:**
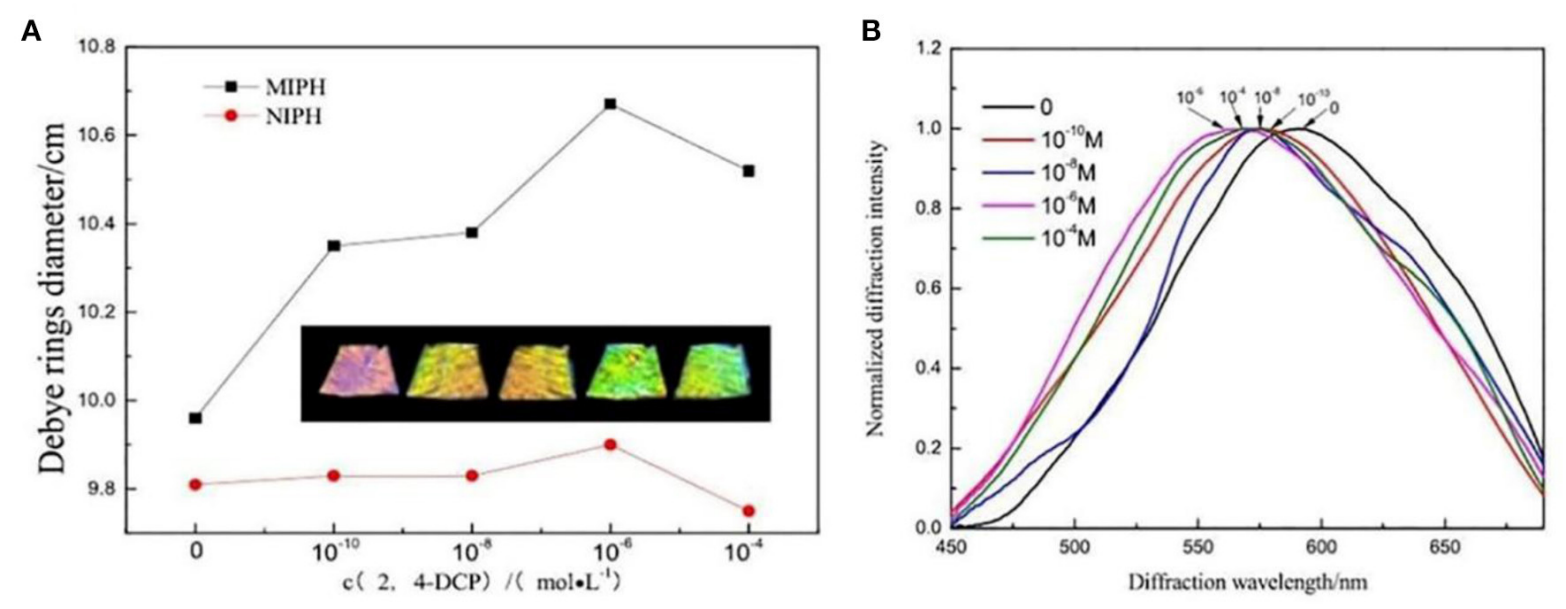
Dependence of **(A)** particle spacing of MIPH/ non-molecularly imprinted two-dimensional photonic crystals hydrogels (NIPH) in 2,4-DCP solution; **(B)** normalized diffraction spectra of MIPH in different concentrations of 2,4-DCP solution (reprinted with the permission of Qin et al., 2020; copyright 2020 Wiley).

Chlorophenols is a kind of antimicrobial, wood preservative, and good pesticide, which has strong bioaccumulation toxicity. The residues of chlorophenols are difficult to degrade and are refractory environmental persistent pollutants. 2,4-Dichlorophenol (2,4-DCP) is a typical pollutant of chlorophenols, and it is necessary to monitor the content of 2,4-DCP to control environmental pollution and ensure human health. Qin et al. (2020) prepared molecularly imprinted two-dimensional PhC hydrogels (MIPH) for sensitive and label-free recognition of 2,4-DCP. The hydrogel was imprinted with 2,4-DCP and the response was monitored through the diameter of Debye diffraction ring. In Figure 6A, the diameter of Debye ring increased by 7.1 mm as the concentration of 2,4-DCP changed from 0 to 1 × 10^−6^ M, and the calculated particle spacing of MIPH reduced 38 nm, which was consistent with the diffraction wavelength shifts in Figure 6B. Meanwhile, the color of MIPH changed from red to green, demonstrating the ability of the naked-eye detection.

Dai et al. (2017a) described a MIP that enabled to detect 2-Butoxyethanol (2BE). 2BE is widely used as a solvent in surface coatings such as lacquers, enamels, varnishes, and latex paint, and it has been identified in hydraulic fracking wastewater in high concentrations, which is considered as an indicator. Compared with NIPs, the MIPs displayed a higher binding capacity for 2BE with imprinting efficiencies of 2. The reflectance wavelength red shifted 50 nm on exposure of the MIPs to 2BE in the concentrations in the range from 1 to 100 ppb. And in the wastewater sample, the MIPs provided a good estimation of the order of magnitude of the concentration of 2BE.

The detection and quantification of cation of metal ions is important in many fields including medical diagnosis, clinical toxicology, environmental monitoring, and waste water management. Schenning (Moirangthem et al., 2016) fabricated a cholesteric liquid crystalline (CLC) polymer benzoic acid metal binding sites to detect Ca^2+^. The chiral imprinted CLC polymer was treated with KOH to turn green. The CLC polymer showed a high response and selectivity for Ca^2+^ ions due to the preorganized binding sites. Especially, the CLC polymer film was sensitive to Ca^2+^ within the physiologically relevant concentration range of 10^−4^–10^−2^ M. Besides the blue shift of the reflection band, the color of the CLC polymer changed from green to blue.

## Application in Human Health

### Proteins and Amino Acids

Proteins are the basis of life and the main undertakers of life activities, which is closely linked with life and all forms of life activities. In addition, amino acids, as the main components of proteins, also play an important role in human life. Currently, proteins have been used as one of the disease markers for the early diagnosis and treatment monitoring of diseases (Wulfkuhle et al., 2003; Morin, 2005; Song et al., 2011; Wu and Qu, 2015). Therefore, it is particularly necessary to detect proteins rapidly and sensitively and develop a quick and convenient method for detecting proteins. Fluorescence (Wang et al., 2010; Chaubard et al., 2012), electrochemical sensors (Xiao et al., 2014), biomass spectrometry (Diamandis, 2004), and immunoassay (Van Den Broek et al., 2013) are mostly used to detect proteins, and all of which need equipment. MIPC sensors have been gradually applied to protein detection due to its convenience, economy, and visual detection.

The health of cerebrospinal fluid (CSF) is a predictor of brain diseases; however, collection of CSF is painful and carries risks (Kuntz et al., 1992; Frost et al., 2010; Inoue et al., 2013). Recently, studies have showed that abnormalities in eyes may indicate an unnatural brain condition. Therefore, diagnosis of a brain disease via a biomarker in the eye could be feasible. Jinn et al. (2019) reported a visually distinguishable light-interfering bio-responsive silica nanoparticle (SNP) embedded avidin imprinted hydrogel sensor. The hydrogel was attached to an intraocular lens (IOL) to detect the abnormalities in the eye by a smartphone application.

**Figure 7:**
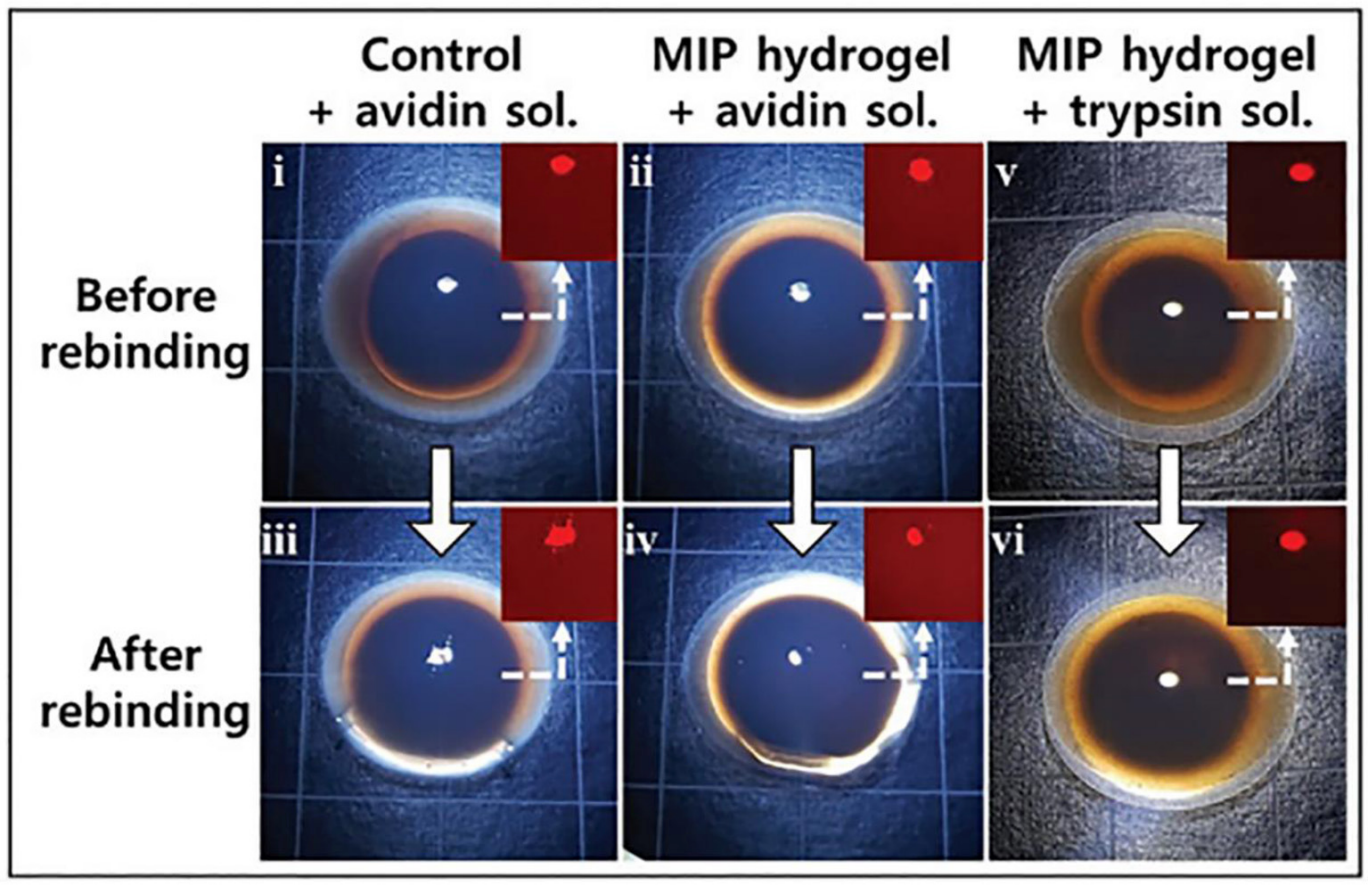
Photographs of light reflected from the hydrogel with various silica nanoparticle concentrations. The insets show an expanded photo of the hydrogel center with only red light remaining (reprinted with the permission of Jinn et al., 2019; copyright 2019 Royal Society of Chemistry).

When avidin is binded to hydrogel, the volume of hydrogel will change, leading to the SNP spacing change. The regular SNP array meets the Bragg's Law and the wavelength of the light shifted with the spacing change, which converted the hydrogel volume change into an amplified optical signal and got a lower detection of limit. The results showed that the volume of avidin-imprinted SNP hydrogel was decreased by 1.56 ± 1.11% and the red-light ratio of reflected light decreased by 3.23 ± 1.01% in 100 nm avidin solution, which were much larger than that of non-imprinted SNP hydrogel of 0.07 ± 1.04 and 1.45 ± 3.83%. Meanwhile, the volume of avidin imprinted hydrogel with another protein solution (trypsin 100 nm) was increased by 0.32 ± 0.62%, and the red-light ratio decreased by 1.02 ± 1.71%. A change in the red light ratio of the reflected light was also observed, and the change was amplified by approximately twice that of the volume change. Representative images of the hydrogels are shown in Figure 7. The molecular-imprinted SNP hydrogel sensor showed high sensitivity and selectivity. Moreover, vitro cell viability and cytotoxicity biocompatibility test has been studied and most survival rates were over 80%. It was confirmed that the generated hydrogel sensor could be applied to an actual IOL.

Pan et al. (2019) reported a molecularly imprinted photonic polymer (MIPP) coated film that detects S-layer proteins (SLP) sensitively and selectively. SLP is a surface protein located on the cell membrane or cell wall of *Lactobacillus* (Frece et al., 2005; Konstantinov et al., 2008), which are related to the adhesion ability of L*actobacillus*, inhibitory effects of the intestinal adhesion of pathogens (Chen et al., 2007; Johnsonhenry et al., 2010), and viral infection (Martínez et al., 2012). The SLP-grafted silica PhC polymer was firstly prepared, and then the SLP-grafted silica was removed to fabricate the inverse opal MIPP. The MIPP showed high sensitivity and selectivity and could reach equilibrium in 3 min. Accordingly, with increasing concentration of SLP in the range from 0 to 1 mg/mL, the MIPP was blue shifted and the LOD was 1 ng/mL. There was a good linear relationship between Bragg shift and SLP concentration, which the linear equation was calculated as Δλ = 1.77X −0.0409, with a correlation coefficient of.9907. Meanwhile, the MIPP was used to analyze real samples. It was observed that MIPP blue shifted in crude extracted SLP diluted 10 times, and the linear fitting equation was fitted, which proved the practical applicability of the MIPP.

Glycoprotein, as a widely distributed protein in the human body, also plays a significant role in biological processes problem and disease warning. Wang H. et al. (2020) presented a novel surface-imprinted inverse opal hydrogel particle functionalized with phenylboronic acid (PBA) for glycoprotein and horseradish peroxidase (HRP) detection. First, silica colloidal crystal beads (SCCBs) were prepared by droplet templates generated from gravity-driven microfluidics and modified with PBA and HRP. After being embedded with gel and washed by HF solution, an inverse opal particle with imprinted sites of HRP was obtained with the imprinting factor of 16.03. When the MIP particles were immersed in HRP solution, HRP molecules accessed the complementary imprinting sites on the hydrogel scaffold and bound with them closely based on the boronated affinity. The results showed that compared with NIP particles, MIP particles were sensitive to HRP and showed high selectivity as immersing in HRP solution with other saccharides. Alpha-fetoprotein (AFP)-imprinted MIP particles with LOD of 1.32 ng/mL were also fabricated to investigate practical applications. Moreover, AFP-imprinted particles were used in human serum samples. And the results fitted with the value quantized by AFP kit.

Chen W. et al. (2019) also reported an imprinted colloidal array for naked-eye detection of glycoproteins. The SNPs were modified with (3-aminopropyl) triethoxysilane (APTES) and 2,4-difluoro-3-formyl PBA (DFFPBA) to obtain DFFPBA-functionalized SNPs (SiO_2_@DFFPBA). HPR-imprinted SiO_2_@DFFPBA/MIP was synthesized according to the boronated affinity-oriented surface imprinting approach, and then HPR was washed out. A close-packed imprinted colloidal array (CPICA) for naked-eye HRP detection was fabricated via a vertical deposition method with SiO_2_@DFFPBA/MIP. The red shifts of reflection peak of the CPICA was 87 nm from 683 to 770 nm as the concentrations of HRP increased from 0 to 25 μm/L, which was obviously more than that of close-packed non-imprinted colloidal array (CPNCA). The selectivity of the CPICA was also studied. The CPICA was immersed in 20 μm/L Lys, OVA, TRF, and BSA, respectively, and the response of the CPICA to HRP was much higher than that of the other proteins, owing to the recognition sites. However, there were no specific binding sites on CPNCA. Only a small amount of protein was bound to CPNCA.

Resende et al. (2020) reported a MIPP sensor-based 3D silica PhC for fibrinopeptide B (FPB) detection, which is a biomarker of venous thromboembolism. Unlike other imprinted sensors, the sensor showed small shifts of the Bragg diffraction with the increase of FPB concentration, but had a significant decrease in the intensity of MIPP reflectance peak, and the decrease of MIPP reflectance peak has a linear relationship with the concentration of FPB. The decrease may be caused by the refractive index change in the presence of FPB. On the contrary, the intensity of NIPP reflectance peak changed irregularly due to the lack of specific recognition cavities tailored for FPB. In synthetic human urine samples, MIPP showed a similar response to FPB, and the LOD was 0.13 ng/mL. The sensor was also used in real human urine samples. The results of standard addition experiment showed that the analysis of samples with concentrations ranging from 0.2 to 22 ng/mL produced accurate data, which suggested that the MIPP sensor may lead to accurate and reproducible readings of FPB in human urine of healthy or diseased individuals.

Amino acids, as the main component of protein, also play an important role in human lives. L-Kynurenine (KYN) is a major metabolite of L-tryptophan (TRP) degradation and is a known potential marker of immune-suppressant disorders and cancer. Rizvi et al. (2020) created a molecularly imprinted hydrogel sensor based on a polystyrene PhC colloidal array (PCCA) for the detection of L-KYN in human serum. The PCCA was made using a needle tip flow method with polystyrene particles of about 629 nm, and the L-KYN was directly imprinted in the hydrogel and washed by acetic acid.

**Figure 8:**
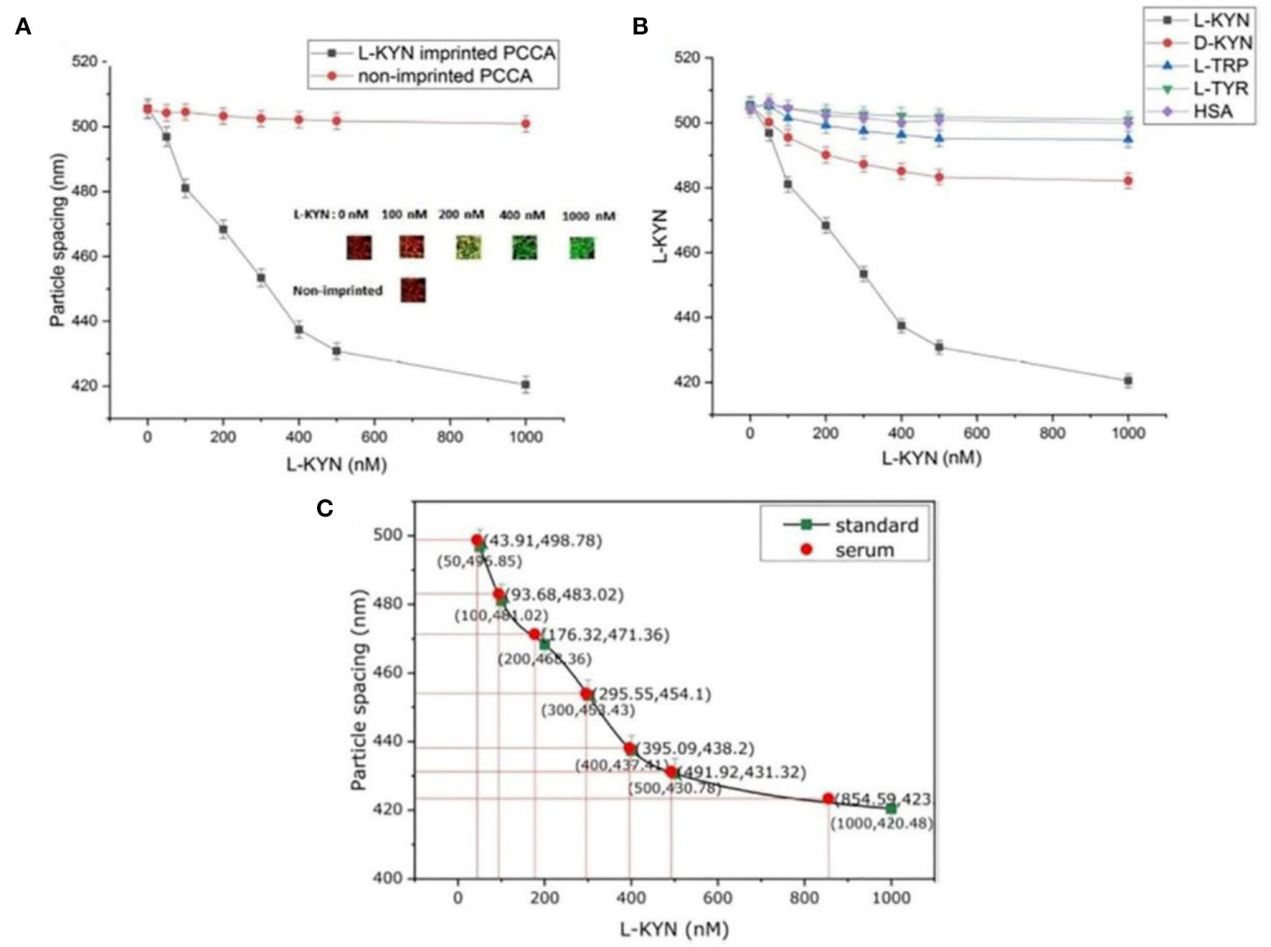
**(A)** Detection of L-KYN by imprinted photonic crystal colloidal array (PCCA) hydrogel; **(B)** selectivity of L-KYN imprinted 2D PCCA hydrogel; and **(C)** determination of L-KYN in human serum (reprinted with the permission of Rizvi et al., 2020; Copyright 2020 Elsevier).

L-KYN-imprinted hydrogel showed a high sensitivity with the LOD of 50 nM, which is 200 times greater than the reported fluorescent sensor. As the L-KYN concentration increased from 50 to 1,000 nM, the particle spacing of imprinted hydrogel decreased from 505 to 420 nm (85 nm) as shown in Figure 8A, with structure color changing from red to green through blue-green. On the contrary, the non-imprinted hydrogel only changed 5 nm with no obvious color change. Meanwhile, selectivity was also studied in Figure 8B. The imprinted hydrogel was immersed in L-KYN, D-KYN, L-TRP, L-tyrosine (L-TRY), and HAS, and the results showed that imprinted hydrogel demonstrated high specificity toward L-KYN. Moreover, to investigate the practical applicability of the L-KYN-imprinted hydrogel, L-KYN in human serum samples was also detected. The serum samples were diluted 10 times and the hydrogel showed a rapid response in a short time of 2 min. The response in human serum samples was compared with that of the standard curve obtained in Figure 8C, which indicated that the hydrogel had similar results in standard L-KYN and human serum.

Chen et al. (2018) described a MIPH to detect L-histidine (L-His). L-histidine (L-His) is an important composition for brain peptides and of muscle proteins (Nan et al., 1999; Rawat and Kailasa, 2014). It has been reported that the under-expression or over-expression of L-His may cause many diseases, like Friedreich ataxia, Parkinson's disease, chronic kidney disease, and psychological disorder (Sasmal et al., 2015). A SiO_2_ PhC array was embedded in the hydrogel with L-His imprinted. Then the array was removed and the imprinted hydrogel film with inverse opal PhCs (IOPCs) structure was obtained. The MIPH could respond rapidly within 60 s and used for five cycles. After optimizing the imprinted conditions, the imprinted hydrogel red shifted 34.1 nm in 100 nM L-His solution and also red shifted 5.8 nm when the concentration of L-His is low to 10 pM. In addition, there were no significant shifts of non-imprinted hydrogel due to no specific recognition sites. These results demonstrate that the MIPH sensor can sensitively detect L-His. The selectivity of MIPH was also studied. MIPH only responded to L-His and in the mix sample, the result was similar to that in L-His, which showed an anti-interference ability to MIPH. And in the drinks sample, the MIPH red shifted and the values of shift are similar to those in phosphate buffer, which indicated the MIPH also had a great performance in practical samples.

### Others

α-Amanitin is the most toxic amanita in mushrooms with lethal dose to humans around 0.1 mg/Kg, so it is important for human health to detect the concentration of α-amanitin. Qiu et al. (2020) reported a molecularly imprinted PhC (MIPC) sensor to detect α-amanitin. The MIPC sensor showed a low LOD of 10^−10^ mg/L, in a short response time of 2 min and good reusability of five cycles. The MIPC sensor red shifted in α-amanitin solution with different concentrations due to its specific-binding sites and non-imprinted PhC (NIPC) showed no response to α-amanitin. The diffraction peak wavelength of MIPC sensor also showed a good linear relationship with the concentration of α-amanitin, and the correlation equation was λ = 15.417c + 489.17 (*R*^2^ = 0.9985) in the range of 10^−9^–10^−3^ mg/L. Moreover, as the wavelength changed, the structure color of MIPC sensor also changed to obtain a naked-eye detection. The response to real samples (mushrooms, urine, and serum) of the MIPC sensors was studied. The MIPC sensors red shifted in spiked α-amanitin solution and the structure color changed from blue to green, which indicated the practical application potential of the MIPC sensors.

Ethyl anthranilate (EA), is one of the main components of grape essence, has also been reported to be used in volatile insect repellent, which was used as an illegal additive for wine. Zhang et al. (2019) prepared a MIPC sensor-based inverse opal SiO_2_ structure to detect EA. Under the optimized conditions, the MIPC showed high sensitivity to EA and the diffraction wavelength shifts of MICA reached 78 nm in 10 mM EA solution. Meanwhile, as the concentration increases, the structure color changed from green to yellow, and finally to red. Thus, the concentration of EA could be observed by different wavelength shifts and structure color change intuitively. The MIPC was also used in actual samples and the wavelength and color of MIPC both red shifted in spiked wine. In addition, the mean recovery of this method was compared with the HPLC method. For the spiked white wine, the mean recovery of MIPC and HPLC are 95.2–103.4 and 96.6–96.4%, respectively, which indicated that MIPC can be extended to routine analysis for EA in real samples.

**Figure 9:**
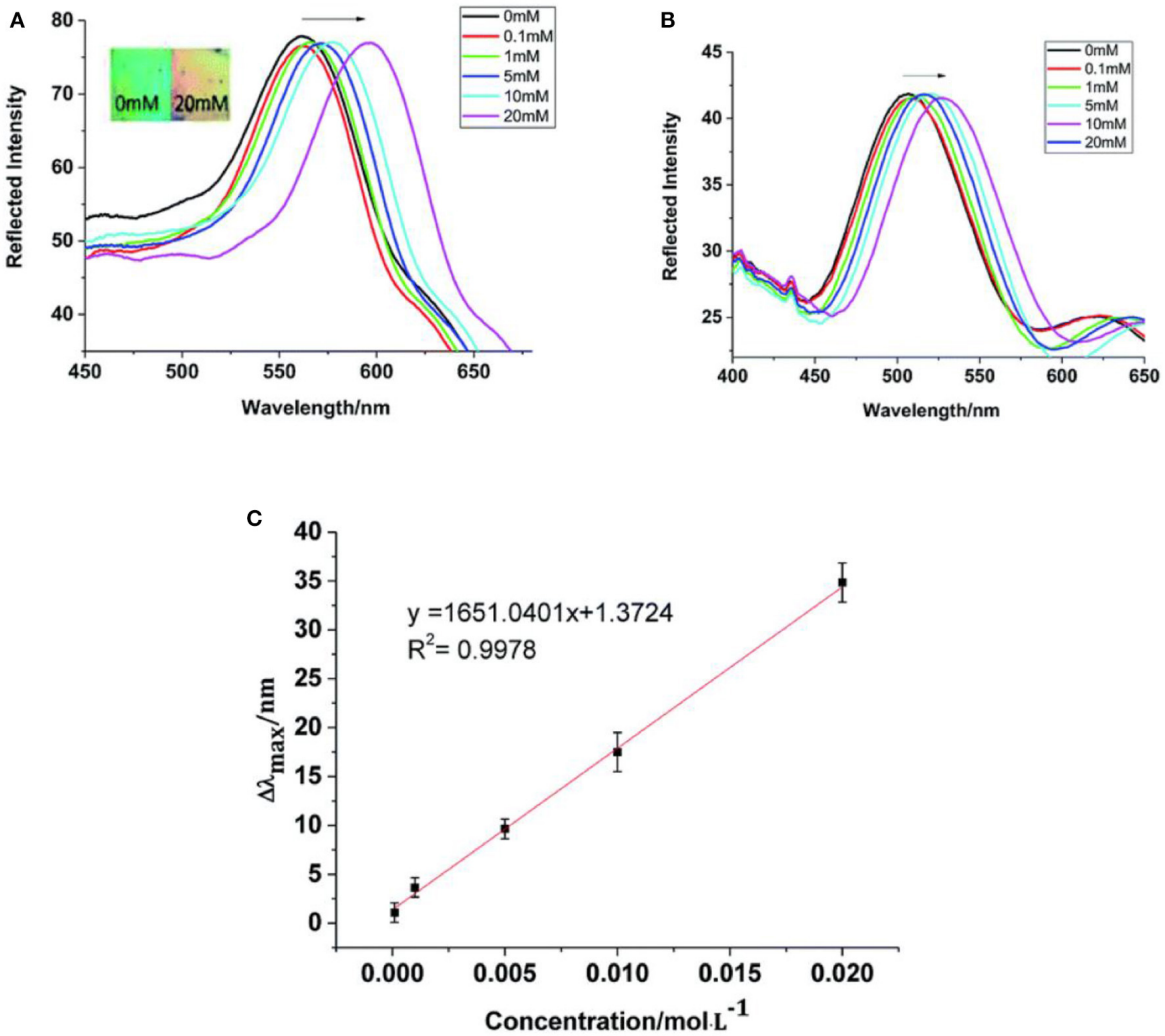
Diffraction spectra of **(A)** molecularly imprinted photonic crystals (MIPC) and **(B)** NIPC samples, and **(C)** a quantitative curve of the MIPC response to benzocaine (reprinted with the permission of Chen S. et al., 2019; copyright 2019 Royal Society of Chemistry).

Chen S. et al. (2019) reported a MIPC hydrogel sensor to detect benzocaine, and the MIPC sensor exhibited high sensitivity, selectivity, rapid response, and good regeneration abilities due to its highly ordered inverse opal structure. Benzocaine is an anesthetic used to improve the survival rates of fish during transportation, which is harmful to humans with excessive intake. The MIPC sensor red shifted with the increase in the concentration of benzocaine and the LOD was 0.1 mM (16.5 mg/mL). In Figure 9, the red-shifts of MIPC reached 35 nm when the concentration was 20 mM and the structure color changed from green to orange. The linear relationship (Δλ_max_ =1.3724 + 1651.0401C, *R*^2^ = 0.9978) between Δλ_max_ and the concentration of benzocaine was also studied. In addition, the MIPC sensor was applied to analyze benzocaine in fish samples and the results were compared with the HPLC method. For the benzocaine at lower concentration, the recovery values of MIPC and HPLC were similar, which indicated that the MIPC can be acceptable and can be extended to the routine analysis of trace benzocaine in real samples.

Testosterone is the primary androgen hormone that has a significant effect on human health. Kadhem et al. (2018) reported a MIP photonic film for the detection of testosterone. The MIP film was based on an inverse opal structure and the pore morphology was about 330 nm. Compared with the NIP, MIP showed high sensitivity and selectivity to testosterone. MIP films were able to detect low concentrations of testosterone from 5 to 100 ppb, and the LOD was 4.2 ppb. And the stability of the MIP films was evidenced by the reversibility of the wavelength shifts observed after six cycles of use and regeneration.

**Figure 10:**
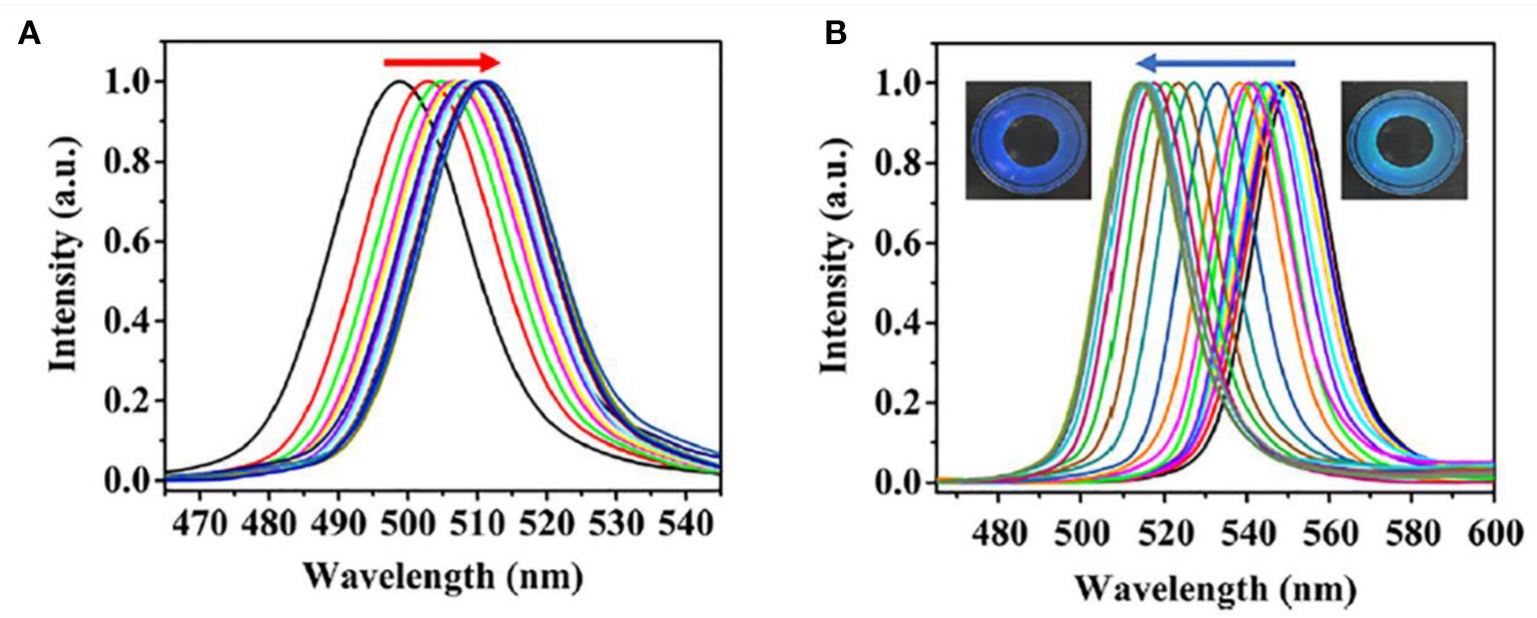
Change of reflection peak of the lens during **(A)** drug loading in pH 6.5 timolol loading solution and **(B)** change of the reflection peak of the lens during drug release in artificial tear fluid (ATF) (reprinted with the permission of Deng et al., 2018; copyright 2018 American Chemistry Society).

Most of the ophthalmic drugs are in the form of eye drops or eye ointments. However, the conventional dosage forms commonly show rapid clearance and low bioavailability (Farandos et al., 2015), and <5% of the drug is effectively absorbed, and lots of the absorbed drug enter the blood circulation and may cause undesirable side effects (Maulvi et al., 2016). In recent years, soft contact lens is considered to be the most promising ophthalmic drug delivery vehicle owing to its unique advantage (Peng et al., 2010). It not only increases the duration of drug residence and bioavailability in the eyes, but is also easy to use and control (Yañez et al., 2011). Deng et al. (2018) proposed a molecular imprinted structural contact lens for sustained timolol release which could self-report the release process by color change, and the results are shown in Figure 10. In the loading timolol section, the reflection peak red shifted 12.4 nm for 12 h due to the combination of timolol and binding sites and the form of the Donnan potential. During the timolol release phase, the interaction between timolol and the monomer gets weaker and timolol is desorbed from the binding sites, which causes the 36.4-nm blue shifts in 12 h.

During timolol loading process, the accumulative mass and reflection peak of the lens all increased over time, and there was a linear relationship between the loading amount and the shifts. Although during timolol release, the reflection peak decreased with the increase of accumulative mass, the relative peak shift was negative, but the absolute value increased linearly with the accumulative releasing amount of timolol. The fascinating contact lens can be further used for controlling the release of a large number of ophthalmic drugs and has high potential to be a new generation of functional contact lenses.

## Conclusion

MIPC sensors have been greatly developed and present great potential in the field of chemical sensing. Herein, we have reviewed the applications in environmental monitoring and human health of MIPC sensors and the responsive mechanisms are also discussed. All the detection by MIPC sensors shows high sensitivity and selectivity with low LOD and short response time. Moreover, real samples analysis is also studied and MIPC sensors present excellent performance, which indicates the unlimited potential in real-time practical applications. However, there are also some limitations of MIPC sensors. (1) The preparation of MIPC requires a large number of organic reagents, which not only has potential harm to human health, but also causes some pollution to the environment. (2) Reported MIPC sensors mainly focus on the single target analyte, and there are still few studies on simultaneous detection of multiple analytes. (3) In addition, due to the influence of imprinting, elution, and detection effects, the variety of target analytes is limited. (4) Due to the limitation of the preparation technology, MIPC sensors are not yet able to achieve large-scale production and cannot be used commercially. Considering the limitations, MIPC sensors will be developed in the following aspects in the future: (1) developing environmentally friendly and green preparation methods of MIPC sensors; (2) maturing preparation process for commercial use as soon as possible; (3) developing high throughput for simultaneous detection of multiple targets; (4) expanding the application of MIPC sensors in other fields, such as microorganisms, and so on. MIPC sensors are not intended to replace existing technologies, but to provide a novel, real-time, and naked-eye detection method. Also, MIPC sensors are still a long way from mass production and commercial use, which also suggests that there still has a lot of room for growth room, and this naked-eye detection sensor is expected to improve in the future.

## Author Contributions

JF: validation, investigation, and writing—original draft. ZM: conceptualization, writing—review and editing, and funding acquisition. XD: formal analysis and software. MX: methodology and conceptualization. LQ: conceptualization, methodology, and funding acquisition. YQ: resources. All authors contributed to the article and approved the submitted version.

## Conflict of Interest

The authors declare that the research was conducted in the absence of any commercial or financial relationships that could be construed as a potential conflict of interest.
